# Migration crisis in Venezuela and its impact on HIV in other countries: the case of Colombia

**DOI:** 10.1186/s12941-019-0310-4

**Published:** 2019-03-08

**Authors:** Alfonso J. Rodríguez-Morales, D. Katterine Bonilla-Aldana, Miguel Morales, José A. Suárez, Ernesto Martínez-Buitrago

**Affiliations:** 1 Annals of Clinical Microbiology and Antimicrobials, Public Health and Infection Research and Incubator Group, Faculty of Health Sciences, Universidad Tecnológica de Pereira, Pereira, Risaralda Colombia; 2 Fundación Universitaria Autónoma de las Américas, Pereira, Risaralda Colombia; 3 Infectious Diseases Organization, Taller Venezolano de VIH, Caracas, DC Venezuela; 4 Investigador SNI Senacyt Panamá, Clinical Research Deparment, Instituto Conmemorativo Gorgas de Estudios de la Salud, Panama City, Panama; 5 Infectious Diseases, Department of Internal Medicine, Universidad del Valle, Santiago de Cali, Colombia

During the last few years, there has been a large migration flux of Venezuelan citizens and refugees. This is a consequence of the current political instability and the economic crisis in that country. Such a situation is leading the migration to countries in South, Central and North America, as well as to Europe, among other regions of the world. This forced displacement is leading also to the importation of infectious diseases as has been recently reported [1–3]. Malaria and other vector-borne diseases [4, 5], tuberculosis, vaccine-preventable diseases [3, 6], among others, such as sexually transmitted diseases and Human Immunodeficiency Virus (HIV) infection. The most direct consequences in public health are to countries of the Americas, which are receiving the massive flux of migration from Venezuela, e.g. Colombia.

Colombia is an example to discuss and enhance the message of the negative consequences of the massive migration from Venezuela and the impact on HIV in a near country. Using and analyzing data from the surveillance system of Colombia, during 2017 (SIVIGILA, https://www.ins.gov.co/Paginas/sistemas-de-informacion.aspx), we explored the incidence of new cases of HIV imported from other countries, particularly including Venezuela.

**Fig. 1:**
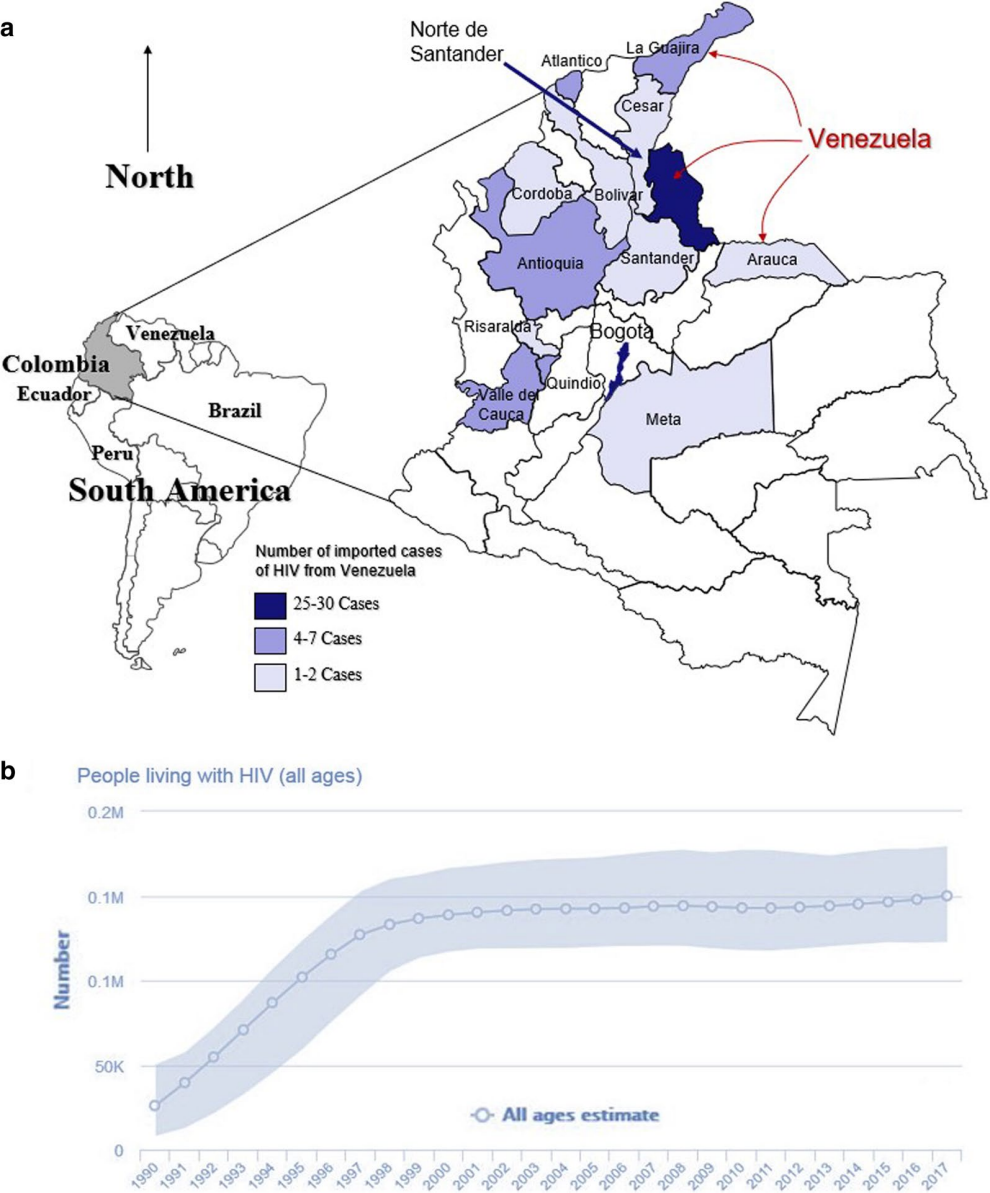
**a** Departments of Colombia receiving imported cases of HIV infection from Venezuela, 2017. **b** Trends in the number of people living with HIV in Colombia, 1990–2017, based on estimates of UNAIDS

In 2017, Colombia reported 13,310 new cases of HIV, with 108 of them imported from other countries (0.8%) [7]. From those imported cases, 83.3% of them (90) were from Venezuela (Table 1). Colombia received newly diagnosed HIV people from 12 other countries. The most affected territory, as expected, was Norte de Santander department, in the border with Venezuela (Fig. 1), followed by the capital of the country, Bogota, and La Guajira department, which is also an international border territory. Norte de Santander reported only 388 autochthonous which means a relation of 1 imported case of Venezuela per 12.9 autochthonous in that territory (Table 1).

**Table 1:** Distribution of imported cases of HIV in Colombia, 2017, according to origin countries and receiving departments of the country

Departments of Colombia	Origin countries														Total	Autochthonous cases in the Department	Ratio Venezuela: autochthonous cases (1 venezuelan per n from Colombia)
	Venezuela	%	Brazil	USA	Spain	Mexico	Peru	Philippines	Israel	Italy	Netherlands	Namibia	Ecuador	Cuba			
Norte de Santander	30	33.3	0	0	0	0	0	0	0	0	0	0	0	0	30	388	12.9
Bogota (Capital)	25	27.8	1	3	1	1	0	1	1	1	1	0	0	0	35	2531	101.2
La Guajira	7	7.8	0	0	0	0	0	0	0	0	0	0	0	0	7	176	25.1
Valle del Cauca	5	5.6	0	0	0	0	0	0	0	0	0	0	0	0	5	1595	319.0
Atlántico (including Barranquilla)	5	5.6	0	0	0	0	0	0	0	0	0	0	0	0	5	816	163.2
Antioquia	4	4.4	0	0	0	0	0	0	0	0	0	0	0	0	4	2108	527.0
Quindío	4	4.4	0	0	0	0	0	0	0	0	0	0	0	0	4	283	70.8
Arauca	2	2.2	0	0	0	0	0	0	0	0	0	0	0	0	2	44	22.0
Bolivar (including Cartagena)	2	2.2	0	0	0	0	0	0	0	0	0	1	0	0	3	578	289.0
Santander	2	2.2	0	0	0	1	0	0	0	0	0	0	0	0	3	472	236.0
Cesar	1	1.1	0	0	0	0	0	0	0	0	0	0	0	0	1	266	266.0
Córdoba	1	1.1	0	0	0	0	0	0	0	0	0	0	0	0	1	763	763.0
Meta	1	1.1	0	0	0	0	0	0	0	0	0	0	0	0	1	185	185.0
Risaralda	1	1.1	0	0	0	0	0	0	0	0	0	0	0	0	1	352	352.0
Amazonas	0	0.0	2	0	0	0	1	0	0	0	0	0	0	0	3	27	–
Cauca	0	0.0	0	0	0	0	0	0	0	0	0	0	1	0	1	219	–
Cundinamarca	0	0.0	0	0	0	0	0	0	0	0	0	0	0	1	1	437	–
Nariño	0	0.0	0	0	1	0	0	0	0	0	0	0	0	0	1	234	–
Total	90	100.0	3	3	2	2	1	1	1	1	1	1	1	1	108	–	–
%	83.3	–	2.8	2.8	1.9	1.9	0.9	0.9	0.9	0.9	0.9	0.9	0.9	0.9	100.0	–	–

Source: National Institute of Health (http://www.ins.gov.co), Colombia and UNAIDS

Countries emerging from a conflict or humanitarian crisis often face conditions that facilitate the spread of HIV, including significant population movements, lack of social and health services in their countries and gender-based violence that leads to these problems. Moving to border countries, where the partnership with humanitarian and assistance organizations is essential to ensure that HIV is adequately addressed in those territories receiving HIV people [8, 9].

During 2017, the Joint United Nations Programme on HIV/AIDS (UNAIDS) estimated that 150,000 adults and children were living with HIV in Colombia, whilst estimated for 2016 there were 120,000 in Venezuela, among whom only 59% were accessing antiretroviral therapy (ART) (only 71,210 people on ART), with only 7% with a low viral load [2, 10]. Over the past decade in Colombia, the number of people living with HIV/AIDS has been stable (Fig. 1) [11]. However, in Venezuela, more than 79,000 people living with HIV stopped receiving antiretrovirals since 2017 and the number of deaths increased from 1800 in 2014 to possibly more than 5000 in 2018. Even more, 154,000 people may be living with HIV in Venezuela, although there are no prevalence and incidence studies with significant coverage [2, 10]. Since 2016, access to ART fell alarmingly, especially due to government lack of funding for it, until it almost disappeared in 2017 and 2018, when international purchases were interrupted leading to approximately 58,000 people with HIV without access to treatment. Given the lack of tests and reagents for it, would impact in a decrease of diagnoses and HIV detection and notification. The impact of such situation even has led to the illegal marketing of ART.

Probably all of it is additionally pressing migration from people of Venezuela with HIV to other countries, including Colombia. In this country, as expected, border territories, such as Norte de Santander and La Guajira, among others, have been significantly impacted by forced migration from Venezuela. A previous report indicated that the number of migrants from Venezuela, significantly increased in the first months of 2017 [12], which is consistent with our findings for this department, but also with impact in other territories of Colombia. Preliminary data of 2018, show that Colombia reported 14,411 new HIV cases during the year, with 135 cases imported from other countries during the first semester of the year, of them 89.6% were from Venezuela, then we can anticipate that at least the number of imported cases in Colombia have doubled for the whole year, most of them from Venezuela.

As has been recently stated by others [3, 6, 10, 13–15], Venezuela is in the midst of an emerging public health crisis, resulting from the collapse of its healthcare system and the re-emergence of previously controlled infectious diseases [14, 16], including HIV, now being receiving in Colombia, Peru as well as in other countries especially in the region of the Americas [1]. On September 27, 2018, the United Nations Human Rights Council adopted a resolution on Venezuela signaling the gravity of the human rights situation and the growing concern by governments worldwide about the country’s humanitarian crisis, including aspects such as malnutrition and the upsurge of preventable diseases. International health organizations [6], including UNAIDS, faces an enormous challenge in attending, without interference, to the complex emergency that affects Venezuela but also migrants, with HIV infection and AIDS. The findings of HIV imported cases from Venezuela in Peru as well as in Colombia, probably are similar in other countries in the Americas, particularly Panama, Ecuador, Peru, Chile, among others, were a significant flux of Venezuelan migrants is occurring, needing more analyses about it. Unpublished data from the Hospital Santo Tomas of Panama City (the largest healthcare center of the Ministry of Health of Panama with 632 beds), show that during 2016–2018, from 2439 new patients diagnosed at the HIV Clinic, 13.5% (329) of them were migrants from Venezuela. In the context of a large shortage of ART, non-rational use practices are reported that have a considerable risk of generating drug resistance and compromising the effectiveness of the treatment, in addition to the risk that the resistance is transmitted and spread to the population level, which is a huge problem, even greater associated with the migration of patients with HIV, who are transmitting a virus with a high probability of resistance to first-line ART in countries with low primary resistance rates, such as Colombia.

With all of the above, it is necessary and a priority that the governments of the countries with the greatest influx of Venezuelan migrants, particularly Colombia, define public health policies in search of evaluating the strategies of detection and prevention of the infection for HIV in this population. Also, specific protocols are necessary to approach and treat patients from Venezuela, given the difficulty of having data from the previous clinical history of them and the unfavorable conditions generated by the abandonment of therapy or late diagnosis.

These protocols must be originated in a combined effort between governments, scientific societies and international supporting organizations, to issue recommendations aimed at providing free testing to refugees, linking those diagnosed or known positive to health care and treat them according to their medical treatment history, if available, guided by resistance testing results or, as a programmatic option, with a regimen that poses low likelihood to be affected by primary or selected resistance associated mutations, such as those based on integrase inhibitors or darunavir/ritonavir, any of both in combination with tenofovir and emtricitabine or lamivudine.
